# Supplementary material for: Usage patterns of aromatherapy essential oil among Chinese consumers
Source: PLoS One. 2022 Aug 15;17(8):e0272031. doi: 10.1371/journal.pone.0272031 (PMC9377617; doi:10.1371/journal.pone.0272031)
Supplement: S1 Appendix — Usage patterns of aromatherapy among the Chinese population who are using essential oils. (PDF) [file pone.0272031.s001.pdf]

Usage patterns of aromatherapy among the Chinese population who are using essential oils

A large survey questionnaire focusing on determining the conditions of essential oils (EOs) used by the Chinese population who are using EOs

* Questions are mandatory and must be completed before submitting.

Section 1 General data (7 questions)

1. What is your gender? *

○Male

○Female

○Prefer not to say

2. What is your age? *

○0-14

○15-24

○25-39

○40-59

○60-70

3. Are you pregnant? *

○Yes

○No

○Maybe

4. What is your occupation? *

○Student

○Self-employed

○Salaried

○Certified aromatherapist

○Housewife

○Retired

○Others

5. What is your body weight? *

○<45 kg

○45-49 kg

○50-54 kg

○55-59 kg

○60-64 kg

○65-69 kg

○≥70 kg

6. What is your body height? *

○<155 cm

○155-159 cm

○160-164 cm

○165-169 cm

○170-174 cm

○175-179 cm

○≥180 cm

7. Which city are you living in now? *

____

Section 2 EOs' consumption (4 questions)

8. Do you use EOs? *

○Yes

○No (Please skip to the end of the questionnaire and submit your answer)

9. For which type of use? (Multiple selection) *

□Inhalation

□Ingestion

□Dermal

□Others

10. Where do you buy your oils? (Multiple selection) *

□Plantation

□Specialized store

□Web store

□Supermarket

□Aromatherapy organization & salon

□Distributors & Suppliers of raw materials for personal care products

□Others (gifts from friends, etc)

11. Who advised you to use EOs? *

○Friends

○Family

○At the point of sale

○Media (TV, Web, Personal media (Weibo, WeChat), etc))

○Aromatherapist

○Beautician

○Others (magazines, books, etc)

Section 3 dermal use (6 questions)

12. Do you use Rose oil? *

○Yes

○No

13. Where do you apply oils on your body? (Multiple selection) *

□Forehead

□Philtrum

□Temples

□Face

□Neck

□Wrists

□Arms

□Breast/chest

□Back

□Shoulders

□Stomach

□Thighs

□Calves/shins

□Feet

□Whole body

14. How often do you use it? *

○Daily _________________
Please write the number of frequencies.

○Weekly _________________
Please write the number of frequencies.

○Monthly _________________
Please write the number of frequencies.

○Yearly _________________
Please write the number of frequencies.

15. How much do you use oil per application? *

○Less than 1% (less than 1 drop in 5 mL base oil)

○1% (1 drop in 5 mL base oil)

○2% (2 drops in 5 mL base oil)

○3% (3 drops in 5 mL base oil)

○4% (4 drops in 5 mL base oil)

○5% (5 drops in 5 mL base oil)

○More than 5% (more than 5 drops in 5 mL base oil)

○Add in cosmetic products _________________
Please write the number of drops.

○Undiluted _________________
Please write the number of drops.

16. Do you use Lavender oil? *

○Yes

○No

17. Where do you apply oils on your body? (Multiple selection) *

□Forehead

□Philtrum

□Temples

□Face

□Neck

□Wrists

□Arms

□Breast/chest

□Back

□Shoulders

□Stomach

□Thighs

□Calves/shins

□Feet

□Whole body

18. How often do you use it? *

○Daily __________________
Please write the number of frequencies.

○Weekly __________________
Please write the number of frequencies.

○Monthly __________________
Please write the number of frequencies.

○Yearly __________________
Please write the number of frequencies.

19. How much do you use oil per application? *

○Less than 1% (less than 1 drop in 5 mL base oil)

○1% (1 drop in 5 mL base oil)

○2% (2 drops in 5 mL base oil)

○3% (3 drops in 5 mL base oil)

○4% (4 drops in 5 mL base oil)

○5% (5 drops in 5 mL base oil)

○More than 5% (more than 5 drops in 5 mL base oil)

○Add in cosmetic products ________________
Please write the number of drops.

○Undiluted ________________
Please write the number of drops.

20. Do you use Tea Tree oil? *

○Yes

○No

21. Where do you apply oils on your body? (Multiple selection) *

□Forehead

□Philtrum

□Temples

□Face

□Neck

□Wrists

□Arms

□Breast/chest

□Back

□Shoulders

□Stomach

□Thighs

□Calves/shins

□Feet

□Whole body

22. How often do you use it? *

○Daily __________________
Please write the number of frequencies.

○Weekly __________________
Please write the number of frequencies.

○Monthly __________________
Please write the number of frequencies.

○Yearly __________________
Please write the number of frequencies.

23. How much do you use oil per application? *

○Less than 1% (less than 1 drop in 5 mL base oil)

○1% (1 drop in 5 mL base oil)

○2% (2 drops in 5 mL base oil)

○3% (3 drops in 5 mL base oil)

○4% (4 drops in 5 mL base oil)

○5% (5 drops in 5 mL base oil)

○More than 5% (more than 5 drops in 5 mL base oil)

○Add in cosmetic products ________________
Please write the number of drops.

○Undiluted ________________
Please write the number of drops.

24. Do you use Ginger oil? *

○Yes

○No

25. Where do you apply oils on your body? *

□Forehead

□Philtrum

□Temples

□Face

□Neck

□Wrists

□Arms

□Breast/chest

□Back

□Shoulders

□Stomach

□Thighs

□Calves/shins

□Feet

□Whole body

26. How often do you use it? *

○Daily _________________
Please write the number of frequencies.

○Weekly _________________
Please write the number of frequencies.

○Monthly _________________
Please write the number of frequencies.

○Yearly _________________
Please write the number of frequencies.

27. How much do you use oil per application? *

○Less than 1% (less than 1 drop in 5 mL base oil)

○1% (1 drop in 5 mL base oil)

○2% (2 drops in 5 mL base oil)

○3% (3 drops in 5 mL base oil)

○4% (4 drops in 5 mL base oil)

○5% (5 drops in 5 mL base oil)

○More than 5% (more than 5 drops in 5 mL base oil)

○Add in cosmetic products _________________
Please write the number of drops.

○Undiluted _________________
Please write the number of drops.

28. Do you use Mint oil? *

○Yes

○No

29. Where do you apply oils on your body? (Multiple selection) *

□Forehead

□Philtrum

□Temples

□Face

□Neck

□Wrists

□Arms

□Breast/chest

□Back

□Shoulders

□Stomach

□Thighs

□Calves/shins

□Feet

□Whole body

30. How often do you use it? *

○Daily ___________________
Please write the number of frequencies.

○Weekly ___________________
Please write the number of frequencies.

○Monthly ___________________
Please write the number of frequencies.

○Yearly ___________________
Please write the number of frequencies.

31. How much do you use oil per application? *

○Less than 1% (less than 1 drop in 5 mL base oil)

○1% (1 drop in 5 mL base oil)

○2% (2 drops in 5 mL base oil)

○3% (3 drops in 5 mL base oil)

○4% (4 drops in 5 mL base oil)

○5% (5 drops in 5 mL base oil)

○More than 5% (more than 5 drops in 5 mL base oil)

○Add in cosmetic products ________________
Please write the number of drops.

○Undiluted ________________
Please write the number of drops.

32. Do you use Lemon oil? *

○Yes

○No

33. Where do you apply oils on your body? (Multiple selection) *

□Forehead

□Philtrum

□Temples

□Face

□Neck

□Wrists

□Arms

□Breast/chest

□Back

□Shoulders

□Stomach

□Thighs

□Calves/shins

□Feet

□Whole body

34. How often do you use it? *

○Daily _________________
Please write the number of frequencies.

○Weekly _________________
Please write the number of frequencies.

○Monthly _________________
Please write the number of frequencies.

○Yearly _________________
Please write the number of frequencies.

35. How much do you use oil per application? *

○Less than 1% (less than 1 drop in 5 mL base oil)

○1% (1 drop in 5 mL base oil)

○2% (2 drops in 5 mL base oil)

○3% (3 drops in 5 mL base oil)

○4% (4 drops in 5 mL base oil)

○5% (5 drops in 5 mL base oil)

○More than 5% (more than 5 drops in 5 mL base oil)

○Add in cosmetic products ________________
Please write the number of drops.

○Undiluted ________________
Please write the number of drops.

36. Do you use Sandalwood oil? *

○Yes

○No

37. Where do you apply oils on your body? (Multiple selection) *

□Forehead

□Philtrum

□Temples

□Face

□Neck

□Wrists

□Arms

□Breast/chest

□Back

□Shoulders

□Stomach

□Thighs

□Calves/shins

□Feet

□Whole body

38. How often do you use it? *

○Daily ________________
Please write the number of frequencies.

○Weekly ________________
Please write the number of frequencies.

○Monthly ________________
Please write the number of frequencies.

○Yearly ________________
Please write the number of frequencies.

39. How much do you use oil per application? *

○Less than 1% (less than 1 drop in 5 mL base oil)

○1% (1 drop in 5 mL base oil)

○2% (2 drops in 5 mL base oil)

○3% (3 drops in 5 mL base oil)

○4% (4 drops in 5 mL base oil)

○5% (5 drops in 5 mL base oil)

○More than 5% (more than 5 drops in 5 mL base oil)

○Add in cosmetic products ________________
Please write the number of drops.

○Undiluted ________________
Please write the number of drops.

40. Do you use Frankincense oil? *

○Yes

○No

41. Where do you apply oils on your body? (Multiple selection) *

□Forehead

□Philtrum

□Temples

□Face

□Neck

□Wrists

□Arms

□Breast/chest

□Back

□Shoulders

□Stomach

□Thighs

□Calves/shins

□Feet

□Whole body

42. How often do you use it? *

○Daily __________________
Please write the number of frequencies.

○Weekly __________________
Please write the number of frequencies.

○Monthly __________________
Please write the number of frequencies.

○Yearly __________________
Please write the number of frequencies.

43. How much do you use oil per application? *

○Less than 1% (less than 1 drop in 5 mL base oil)

○1% (1 drop in 5 mL base oil)

○2% (2 drops in 5 mL base oil)

○3% (3 drops in 5 mL base oil)

○4% (4 drops in 5 mL base oil)

○5% (5 drops in 5 mL base oil)

○More than 5% (more than 5 drops in 5 mL base oil)

○Add in cosmetic products __________________
Please write the number of drops.

○Undiluted __________________
Please write the number of drops.

44. Do you use Ylang ylang oil? *

○Yes

○No

45. Where do you apply oils on your body? (Multiple selection) *

□Forehead

□Philtrum

□Temples

□Face

□Neck

□Wrists

□Arms

□Breast/chest

□Back

□Shoulders

□Stomach

□Thighs

□Calves/shins

□Feet

□Whole body

46. How often do you use it? *

○Daily _________________
Please write the number of frequencies.

○Weekly _________________
Please write the number of frequencies.

○Monthly _________________
Please write the number of frequencies.

○Yearly _________________
Please write the number of frequencies.

47. How much do you use oil per application? *

○Less than 1% (less than 1 drop in 5 mL base oil)

○1% (1 drop in 5 mL base oil)

○2% (2 drops in 5 mL base oil)

○3% (3 drops in 5 mL base oil)

○4% (4 drops in 5 mL base oil)

○5% (5 drops in 5 mL base oil)

○More than 5% (more than 5 drops in 5 mL base oil)

○Add in cosmetic products _________________
Please write the number of drops.

○Undiluted _________________
Please write the number of drops.

48. Do you use Eucalyptus oil? *

○Yes

○No

49. Where do you apply oils on your body? (Multiple selection) *

□Forehead

□Philtrum

□Temples

□Face

□Neck

□Wrists

□Arms

□Breast/chest

□Back

□Shoulders

□Stomach

□Thighs

□Calves/shins

□Feet

□Whole body

50. How often do you use it? *

○Daily __________________
Please write the number of frequencies.

○Weekly __________________
Please write the number of frequencies.

○Monthly __________________
Please write the number of frequencies.

○Yearly __________________
Please write the number of frequencies.

51. How much do you use oil per application? *

○Less than 1% (less than 1 drop in 5 mL base oil)

○1% (1 drop in 5 mL base oil)

○2% (2 drops in 5 mL base oil)

○3% (3 drops in 5 mL base oil)

○4% (4 drops in 5 mL base oil)

○5% (5 drops in 5 mL base oil)

○More than 5% (more than 5 drops in 5 mL base oil)

○Add in cosmetic products __________________
Please write the number of drops.

○Undiluted __________________
Please write the number of drops.

52. Do you use Jasmine oil? *

○Yes

○No

53. Where do you apply oils on your body? (Multiple selection) *

□Forehead

□Philtrum

□Temples

□Face

□Neck

□Wrists

□Arms

□Breast/chest

□Back

□Shoulders

□Stomach

□Thighs

□Calves/shins

□Feet

□Whole body

54. How often do you use it? *

○Daily __________________
Please write the number of frequencies.

○Weekly __________________
Please write the number of frequencies.

○Monthly __________________
Please write the number of frequencies.

○Yearly __________________
Please write the number of frequencies.

55. How much do you use oil per application? *

○Less than 1% (less than 1 drop in 5 mL base oil)

○1% (1 drop in 5 mL base oil)

○2% (2 drops in 5 mL base oil)

○3% (3 drops in 5 mL base oil)

○4% (4 drops in 5 mL base oil)

○5% (5 drops in 5 mL base oil)

○More than 5% (more than 5 drops in 5 mL base oil)

○Add in cosmetic products ________________
Please write the number of drops.

○Undiluted ________________
Please write the number of drops.

56. Do you use other types of EO absent from above list?
Please write other types of EO absent from our list.

____
