# Supplementary material for: Usage patterns of aromatherapy essential oil among Chinese consumers
Source: PLoS One. 2022 Aug 15;17(8):e0272031. doi: 10.1371/journal.pone.0272031 (PMC9377617; doi:10.1371/journal.pone.0272031)
Supplement: S1 Table — (PDF) [file pone.0272031.s002.pdf]

**S1 Table. Demographical characteristics of the total consumers.**

| *Number of consumers related to the total consumers/Percentage* | *Total* | *Male* | *Female* |
|---|---|---|---|
| | N = 534 (100%) | N = 53 (9.90%) | N = 481 (90.10%) |
| 0–14 | N = 18 (3.37%) | N = 8 (15.09%) | N = 10 (2.08%) |
| 15–24 | N = 27 (5.06%) | N = 5 (9.43%) | N = 22 (4.57%) |
| 25–39 | N = 321 (60.11%) | N = 16 (30.19%) | N = 305 (63.41%) |
| 40–59 | N = 159 (29.78%) | N = 21 (39.62%) | N = 138 (28.69%) |
| 60–70 | N = 9 (1.69%) | N = 3 (5.66%) | N = 6 (1.25%) |
| Pregnancy | N = 20 (3.74%) | | N = 20 (4.16%) |
| None pregnancy | N = 514 (96.26%) | N = 53 (100%) | N = 461 (95.84%) |
| Student | N = 43 (8.05%) | N = 13 (24.52%) | N = 30 (6.24%) |
| Self-employed | N = 68 (12.73%) | N = 13 (24.52%) | N = 55 (11.43%) |
| Salaried | N = 166 (31.08%) | N = 18 (33.96%) | N = 168 (30.77%) |
| Aromatherapist | N = 69 (12.92%) | N = 1 (1.89%) | N = 68 (14.14%) |
| Housewife | N = 93 (17.40%) | | N = 93 (19.33%) |
| Retired | N = 16 (3.00%) | N = 3 (5.66%) | N = 13 (2.70%) |
| Others (civil servant, etc) | N = 79 (14.79%) | N = 5 (9.43%) | N = 74 (15.38%) |
| East | N = 459 (85.96%) | N = 36 (67.92%) | N = 423 (87.94%) |
| Central | N = 49 (9.18%) | N = 10 (18.87%) | N = 39 (8.11%) |
| Northeast | N = 5 (0.94%) | N = 1 (1.89%) | N = 4 (0.83%) |
| West | N = 21 (3.93%) | N = 6 (11.32%) | N = 15 (3.12%) |
