# Supplementary material for: Usage patterns of aromatherapy essential oil among Chinese consumers
Source: PLoS One. 2022 Aug 15;17(8):e0272031. doi: 10.1371/journal.pone.0272031 (PMC9377617; doi:10.1371/journal.pone.0272031)
Supplement: S1 Fig — (PDF) [file pone.0272031.s003.pdf]

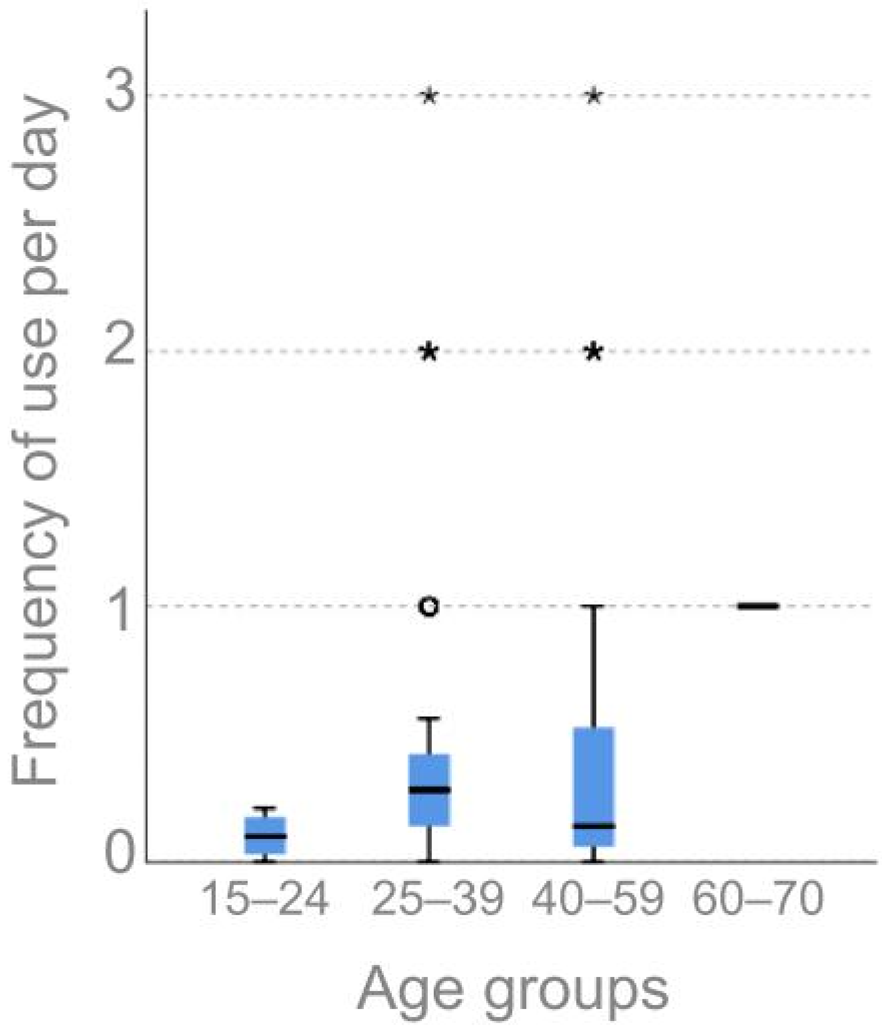

S1 Fig. Use frequency of Ginger oil across age groups. Result expressed in day-1: difference on use frequency of females using Ginger oil was determined for 4 age groups with a Kruskall-Wallis test. Notably, females aged 0-14 do not use Ginger oil.
