# Supplementary material for: Usage patterns of aromatherapy essential oil among Chinese consumers
Source: PLoS One. 2022 Aug 15;17(8):e0272031. doi: 10.1371/journal.pone.0272031 (PMC9377617; doi:10.1371/journal.pone.0272031)
Supplement: Attachment — Submitted filename: rebuttal letter.docx [file pone.0272031.s004.docx]

Dear Editors and Reviewers:

Thank you for your letter and for the reviewers’ comments concerning our manuscript entitled “Usage Patterns of Aromatherapy Essential Oil Among Chinese Consumers” (ID: PONE-D-21-39234). Those comments are all valuable and very helpful for revising and improving our paper, as well as the important guiding significance to our researches. We have studied comments carefully and have made correction which we hope meet with approval. Revised portion are marked in red in the paper. The main corrections in the paper and the responds to the reviewer’s comments are as flowing:

Responds to the reviewer’s comments:

Reviewer #1:

1. **Comment:** Current manuscript give a good account about the usage pattern of aromatherapy essential oil among Chinese consumers. Table 1, present the number and percentage of users through dermal routs per age groups. if possible to add in the first row of the table Frequency: n(%) instead of repeating the letter N in every row. also, Chi-square should be applied between different age group, at each gender, to evaluate the significance either within or between groups. an overall comment, table 1 should be re-disgned or reformatted to better express your results. table 2, please add the significance and test used in the table.

**Response:** It is really true as Reviewer suggested that Table 1 should be re-designed. We re-designed Table 1 in Line 185.

It is reasonable to use Chi-square test to compare the differences between different age groups, at each gender. And we tried to use Chi-square test to evaluate the significance between or within different age groups at each gender, however, since the female dermal users were overrepresented (N = 457; 92.5% of total dermal users), and even within female dermal users, due to small sample size of female aged 0–14 and 60–70, the results of evaluation seems meaningless. Thus, the results are not shown in this manuscript.

Considering Reviewer’s suggestion on adding the significance and test used in the table, we added the Kruskall-Wallis test in the Table footnotes belong to Table 2 in Line 257-259.

2. **Comment:** materials and methods doesn't include details about the data, either parametric or non parametric, what statistical analysis was used, to evaluate or assess what.

**Response:** We are very sorry for our negligence of giving details on the data, as well as statistical analysis used. Thus, in revised manuscript, we have made correction according to the Reviewer’s comments.

Line 141-143, “Data on the type of EOs, exposed body areas, dilution rates of use are treated as non-parametric. And data on frequency of use for each EO is treated as parametric. ” were added.

Line 143-145, the statements of “The chi-squared or Fisher’s test was conducted to compare the differences in percentages of users per age groups.” were corrected as “The chi-squared or Fisher’s test was conducted to compare the differences in percentages of users on the type of EOs, exposed body area, and dilution rates of use across age groups. ”

Line 153-155, the statements of “The Kruskall-Wallis test was conducted to compare frequencies per age and per type of EO among females.” were corrected as “The Kruskall-Wallis test was conducted to compare difference in frequencies of use per age and per type of EO among females.”

3. **Comment:** also, sample size should presented in the materials and methods part, how many samples (participants) how sample size was calculated and a reference should be included?

**Response:** It is really true as Reviewer suggested that we should explain the sample size in the material and methods parts, thus we corrected it as following:

Line 79, “ there are approximately 1,500 EO consumers registered to share” were added to explain the potential sample size.

Special thanks to you for your good comments.

Reviewer #2:

1. **Comment:** The study presents the results of original research. The article needs some revision to be suitable for publication. The general grammar should be revised to enhance clarity and enriched with recent references (2016-2022).

**Response:** As Reviewer suggested, we revised manuscript to fully meet PLOS ONE’s publication criteria, and we also added three recent references which were published in 2019, 2018, and 2021, respectively in Line 45: [8, 9] and Line 343: [32].

We appreciate for Editors and Reviewers’ warm work earnestly, and hope that the correction will meet with approval. Once again, thank you very much for your comments and suggestions
